# Neurofibromatosis in a 16 Year old

**Published:** 2013-01-21

**Authors:** Michael J. Ingargiola, Ian C. Hoppe, Mark S. Granick

**Affiliations:** Division of Plastic Surgery, Department of Surgery, New Jersey Medical School—University of Medicine and Dentistry of New Jersey, Newark

**Figure:**
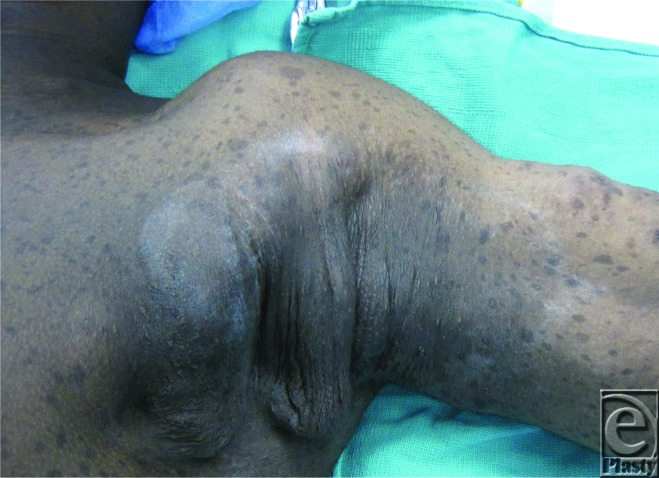


## DESCRIPTION

A 16-year-old adolescent boy with neurofibromatosis presents with neurofibromas of the axilla and arm.

## QUESTIONS

1. **What is the etiology of these lesions?**
2. **What other clinical findings might be seen in this disease?**
3. **What are the management options?**

## DISCUSSION

Neurofibromatosis (NF) type 1, an autosomal-dominant disorder, is caused by a mutation in the neurofibromin gene, which is located on chromosome 17. The gene encodes a tumor suppressor that has roles in inhibiting the *ras* pathway through intrinsic GTPase activation.^1^ This mutation results in development of tumors of the peripheral nervous system, neurofibromas, and specifically in the peripheral nerve sheath. Neurofibromas, categorized as dermal or plexiform, are a major diagnostic criteria for this disease. Dermal neurofibromas, benign growths at small nerve endings or large branches, can develop in high numbers and be painful to the patient. Plexiform neurofibroma, benign growth in the nerve sheath that can run the length of multiple nerves, presents more problem for the patient, with more debilitating effects and the potential for malignant degeneration.^2^ Neurofibromas can cause thickening of the nerve and lead to soft tissue hypertrophy. Because of the melanocyte involvement, skin above the affected area may be hyperpigmented. This explains the café au lait spots that are commonly seen in this condition. These growths can occur at any time throughout life and are often seen in early childhood and times of hormonal change.^3^ Other clinical signs that are seen in these patients are defined by the NIH criteria for NF type 1 (Table).

The criteria are met in an individual if 2 or more of the features listed are present. This Table has been taken from http://www.medicalcriteria.com/criteria/neuro_nf.htm (cited June 5, 2012).

Many problems are associated with the development of these tumors that necessitate close monitoring in the patient with NF. Neurologic symptoms, musculoskeletal abnormalities, with some of the most common being unilateral dysplastic appearance of the greater wing of the sphenoid, scoliosis, and thoracic spine kyphosis, and manifestations involving the cardiovascular, pulmonary, and gastrointestinal systems, may be present along with the commonly seen dermatological symptoms. Development of malignant tumors is more likely in these patients. The transformation of a plexiform neurofibroma to a malignant peripheral nerve sheath tumor is relatively rare but very aggressive.^4^

Resection of these tumors is the most common method of treatment, but it presents numerous problems. Because of the tumors' location on multiple fascicles of the peripheral nervous system, the risk of nerve damage leading to neurological and functional damage is quite high. Another problem is that recurrence of the tumor following resection has shown to be very common. For these reasons, surgery is often postponed until malignant suspicion or the tumor causes other detrimental symptoms. However, removal of early superficial plexiform neurofibromas has shown to decrease the recurrence rate, slow or stop hypertrophy, and decrease development of some of the more worrisome outcomes.^5^ Surgery appears to be the management of choice for these tumors, especially the plexiform neurofibromas. Various chemotherapies are being investigated and supposed to provide a safer and more effective treatment method. Recently, attempts have been made to target the specific Schwann cells or fibroblasts involved with the tumor growth.^6^ CO_2_ laser therapy is a viable option for patients with many small dermal neurofibromas, although it has been shown that this method carries with it an increased potential for the development of hypertrophic scars.^7^

## Figures and Tables

**Table:** NIH diagnostic criteria for neurofibromatosis

Diagnosis of Neurofibromatosis Type 1 (NF1)	
1.	Six or more café au lait macules more than 5 mm in greatest diameter in prepubertal individuals and more than 15 mm in greatest diameter in postpubertal individuals
2.	Two or more neurofibromas of any type or one plexiform neurofibroma
3.	Freckling in the axillary or inguinal regions (Crowe's sign)
4.	Optic glioma
5.	Two or more Lisch nodules (iris hamartomas)
6.	A distinctive osseous lesion such as sphenoid dysplasia or thinning of long bone cortex with or without pseudoarthrosis
7.	A first-degree relative (parent, sibling, or offspring) with NF1 by the criteria mentioned earlier
